# The Genetic Architecture of Ovariole Number in *Drosophila melanogaster*: Genes with Major, Quantitative, and Pleiotropic Effects

**DOI:** 10.1534/g3.117.042390

**Published:** 2017-05-26

**Authors:** Amanda S. Lobell, Rachel R. Kaspari, Yazmin L. Serrano Negron, Susan T. Harbison

**Affiliations:** Laboratory of Systems Genetics, National Heart Lung and Blood Institute, National Institutes of Health, Bethesda, Maryland 20892

**Keywords:** ovariole number, sleep, genome-wide association study, *Drosophila melanogaster*

## Abstract

Ovariole number has a direct role in the number of eggs produced by an insect, suggesting that it is a key morphological fitness trait. Many studies have documented the variability of ovariole number and its relationship to other fitness and life-history traits in natural populations of *Drosophila*. However, the genes contributing to this variability are largely unknown. Here, we conducted a genome-wide association study of ovariole number in a natural population of flies. Using mutations and RNAi-mediated knockdown, we confirmed the effects of 24 candidate genes on ovariole number, including a novel gene, *anneboleyn* (formerly *CG32000*), that impacts both ovariole morphology and numbers of offspring produced. We also identified pleiotropic genes between ovariole number traits and sleep and activity behavior. While few polymorphisms overlapped between sleep parameters and ovariole number, 39 candidate genes were nevertheless in common. We verified the effects of seven genes on both ovariole number and sleep: *bin3*, *blot*, *CG42389*, *kirre*, *slim*, *VAChT*, and *zfh1*. Linkage disequilibrium among the polymorphisms in these common genes was low, suggesting that these polymorphisms may evolve independently.

Ovariole number in insects is a quantitative trait that affects reproductive fitness by impacting the number of eggs produced by a female fly (Bouletreau-Merle *et al.* 1982; R’kha *et al.* 1997; Klepsatel *et al.* 2013b). Ovariole number is related to egg production at different times during the lifespan, with a greater correlation during early life than at later ages (Schmidt *et al.* 2005). Ovariole number exhibits latitudinal clines (Capy *et al.* 1993; Azevedo *et al.* 1996; Schmidt *et al.* 2005), suggesting the influence of natural selection. Importantly, variation in ovariole number has a significant genetic component (Robertson 1957; Wayne *et al.* 1997, 2001; Wayne and Mackay 1998; Orgogozo *et al.* 2006; Bergland *et al.* 2008), which is therefore subject to natural selection. Candidate genes for ovariole number have been identified and include *bab1*, genes in the *Hippo* pathway, and *InR* (Orgogozo *et al.* 2006; Green and Extavour 2012, 2014; Sarikaya *et al.* 2012; Sarikaya and Extavour 2015). But the extent of genes that contribute to genetic variability in ovariole number in natural populations is unknown.

Here, we measured ovariole number traits in the *Drosophila* Genetic Reference Panel (DGRP). We measured ovariole number and ovariole asymmetry, and calculated the coefficient of environmental variation (*CV*_E_) for these two traits as well. With the exception of ovariole asymmetry *CV*_E_, we found high levels of genetic variation among DGRP lines for ovariole number related traits, suggesting that they could be mapped by association analysis. Genome-wide association (GWA) implicated 89 candidate genes for ovariole number, 115 for ovariole number asymmetry, and 769 for ovariole number *CV*_E_. Using *Minos*/*P*-element insertions and RNAi-mediated knockdown, we confirmed 24 candidate genes for ovariole number. Many of these constructs had quantitative effects, changing as much as 4.6 ovarioles with a single mutation. But we also observed major effects on ovariole number when two candidate genes were perturbed. A *Minos* insertion in one of the candidate genes, *anneboleyn* (formerly *CG32000*), had large, qualitative effects on ovariole morphology. *anneboleyn* females also produced fewer offspring. Our analysis implicated *zfh1* as well, a gene with known effects on ovariole number development (Moore *et al.* 1998). Thus, the GWA identified genes with both subtle, quantitative effects on ovariole number as well as gross morphological defects.

To discover potential evolutionary trade-offs, ovariole number has been contrasted with other life-history traits. For example, ovariole number is correlated with body size across insect species (Honek 1993) and in *Drosophila melanogaster* (Bergland *et al.* 2008). Here, we correlated ovariole number phenotypes with sleep, and we explored potential pleiotropic effects between candidate genes for ovariole number and sleep by comparing our GWA results to a previous study of sleep in the same population of flies (Harbison *et al.* 2013). Neither ovariole number nor ovariole asymmetry were genetically correlated with sleep, though genetic correlations were found among the coefficients of environmental variation for these traits. Furthermore, the overlap among polymorphic variants impacting the two suites of traits was low. Nevertheless, 344 genes were common to both studies, with 39 genes overlapping between ovariole number and sleep. Effects on sleep and ovariole number were confirmed for seven genes. Linkage disequilibrium (LD) patterns in genes common to both studies suggest independent evolution of the two suites of traits.

Thus, ovariole number is a typical quantitative trait affected by many genes of small effect as well as several large-effect genes. Despite its role as a fundamental morphological trait, ovariole number genes exhibit pleiotropy with sleep, implying a complex relationship with natural selection and fitness.

## Materials and Methods

### Ovariole phenotypes

Ovariole number and asymmetry were measured in the DGRP—a collection of 205 inbred lines generated through 20 generations of full-sib mating of progeny of *D. melanogaster* females wild-caught in Raleigh, North Carolina (Mackay *et al.* 2012; Huang *et al.* 2014). Flies were maintained under standard culture conditions (cornmeal-sucrose medium, 25°, 50–60% relative humidity) with a 12-hr light-dark cycle. For each DGRP line, we seeded two parental cultures with five male flies and five female flies to control parental density. Parents were cleared from culture vials after 6 d, before offspring eclosion. Six days after eclosion, we transferred adult progeny to vials containing standard medium plus a dab of thick yeast paste. The addition of yeast paste encourages egg development to facilitate ovariole counting (Bergland *et al.* 2008). After 5 d on the yeast paste medium, female flies were frozen at −20°. A pilot study revealed that counting ovarioles from 10 female flies per DGRP line gave 80% power to detect a four ovariole-difference at *α* = 0.05 (Sokal and Rohlf 1995). Based on this power calculation, we counted ovariole number in both ovaries of 10 female flies per DGRP line, and replicated these measures three times, resulting in the measurements of 30 flies per DGRP line. Ovarioles were counted within 1 month of freezing. Ovaries were dissected under magnification in a drop of PBS, and stained with a saturated solution of crystal violet (Fisher Scientific, Hampton, NH), which facilitated the identification and counting of individual ovarioles.

The ovariole number of each fly was defined as the mean of the number of ovarioles in the right and left ovaries. Ovariole asymmetry was defined as the absolute value of the difference in ovariole number between the right and left ovaries. Ovariole asymmetry is sometimes calculated as the percent difference in ovariole number. We found that this percentage scaled linearly with asymmetry (*r* = 0.779), and thus did not examine it as a separate quantitative trait. In addition, for each DGRP line, the coefficient of environmental variation (*CV*_E_) for ovariole number and asymmetry was calculated as (*σ*_E_/*μ*) × 100, where *µ* is the mean trait value per replicate, and *σ*_E_ is the within-replicate SD (Mackay and Lyman 2005).

### Quantitative genetic analyses

We partitioned the variance in ovariole number and ovariole asymmetry using the ANOVA model *Y* = *μ* + *R* + *L* + *R*×*L* + *V*(*R*×*L*) + *ε*, where *R* (replicate), *L* (line), and *V* (vial) are random effects, and *ε* is the error variance. In this model, *Y* is the phenotype; *μ* is the overall trait mean; *R* is the replicate effect; *L* is the main effect of DGRP line (*i.e.*, the genetic component of variance); *L*×*R* accounts for the interaction of each line with the environmentally derived variance across the three experimental replicates; and *V*(*R*×*L*) accounts for environmental variance derived from a fly being reared in a particular culture vial, including potential effects due to differences in rearing density. Broad sense heritability (*H*^2^) of ovariole number and asymmetry was calculated as *H*^2^ = *σ*^2^_L_/(*σ*^2^_R_ + *σ*^2^_L_ + *σ*^2^_R×L_ + *σ*^2^_V(R×L)_ + *σ*^2^_ε_), where *σ*^2^_L_ is the variance component among lines, and *σ*^2^_R_, *σ*^2^_R×L_, *σ*^2^_V(R×L)_, and *σ*^2^_ε_ are all other potential sources of variance in the model.

We partitioned the variance in ovariole number *CV_E_* and ovariole asymmetry *CV_E_* using the ANOVA model *Y* = µ + *L* + *ε*, where *Y* is the phenotype, *μ* is the overall *CV*_E_ mean, *L* is the random effect of DGRP line, and *ε* is the error term. Broad sense heritability (*H*^2^) of *CV_E_* was calculated as *H*^2^ = *σ*^2^_L_/(*σ*^2^_L_ + *σ*^2^_ε_), where *σ*^2^_L_ is the variance component among lines, and *σ*^2^_ε_ are the remaining potential sources of variance in the model.

Phenotypic correlations (*r*_P_) between each reproductive trait, and between reproductive traits and female values for 14 previously measured sleep phenotypes (Harbison *et al.* 2013), were calculated using Pearson’s correlation coefficient in the software package JMP 13.0.0 (SAS Institute, Cary, NC). Genetic correlations (*r*_G_) between traits were calculated as *cov*_12_/√(*σ_L1_^2^* × *σ_L2_^2^*) (Falconer and Mackay 1996), where *cov*_12_ is the covariance between traits 1 and 2, and *σ_L1_^2^* and *σ_L2_^2^* are the among-line variances for traits 1 and 2, respectively. Except for the phenotypic correlations, all other quantitative genetic analyses were conducted using SAS software (SAS Institute).

### Genotype/phenotype associations

We associated mean ovariole number, ovariole asymmetry, and ovariole number *CV*_E_ for each DGRP line with genetic variation segregating in the DGRP using three separate approaches. In each approach, we associated the 3,461,238 sites segregating in the DGRP having a minor allele frequency (MAF) ≥0.01 with ovariole traits. In the first approach, we used the Factored Spectrally Transformed Linear Mixed Model (FaST-LMM) (Lippert *et al.* 2011) for GWA, and adjusted the model for genetic relatedness among the DGRP lines. We used the DGRP Freeze 2.0 analysis pipeline available online (http://dgrp2.gnets.ncsu.edu) to obtain ovariole phenotypes adjusted for the presence/absence of five common chromosomal inversions and *Wolbachia pipientsis* infection status, and incorporated these phenotypes into the FaST-LMM model (Huang *et al.* 2014). Note that, in order to increase computational speed, FaST-LMM uses mean imputation to determine the genotype of variants with missing calls (Lippert *et al.* 2011). In the second approach, we used FaST-LMM to associate polymorphisms with adjusted ovariole traits, but without adjusting the model for genetic relatedness among the DGRP lines. This enabled us to contrast the effect of incorporating genetic relatedness. In the third approach, we applied a general linear model (GLM) *Y* = μ + *M* + *ε*, where *Y* is the phenotype, *μ* is the trait mean, *M* is variant genotype, and *ε* is the error term; in this approach, we did not adjust ovariole number phenotypes, nor correct for population structure. The GLM approach allowed us to evaluate the effects of phenotypic adjustment as well as imputation. Genetic variants significantly associated with ovariole phenotypes were defined as those with a nominal discovery *P*-value of ≤1 × 10^−5^ in any of the three approaches, consistent with previous studies using the DGRP (Arya *et al.* 2015; Dembeck *et al.* 2015a,b; Garlapow *et al.* 2015; Shorter *et al.* 2015; Zwarts *et al.* 2015; Hunter *et al.* 2016). We also calculated the false discovery rate (FDR) for each association using the Benjamini-Hochberg procedure (Benjamini and Hochberg 1995). The GLM and FDR calculations were performed using SAS software (SAS Institute). For each trait, variant effect sizes (*a*) were calculated as one-half the difference in mean trait value between all DGRP lines carrying the minor allele and those carrying the major allele at that variant position (Falconer and Mackay 1996). Standardized effect sizes (*a*/*σ*_G_) were calculated as variant effect sizes divided by the SD of genetic variation in the trait across the DGRP. Minor allele frequencies were calculated as the quotient of the number of DGRP lines carrying the minor allele at a given locus divided by the total number of DGPR lines with known genotypes at the locus. Effect sizes and allele frequencies were calculated using JMP 13.0.0 (SAS Institute). LD among SNPs was computed using PLINK 1.07 (Purcell *et al.* 2007).

### Verification of genotype–phenotype associations

GWA analyses implicated variants for ovariole number that fell in, or within, 1 kb of 89 putative candidate genes. In 29 instances, polymorphic variants mapped to two overlapping genes; in one instance, the variant mapped to three overlapping genes. As the FDRs for GWA variants for ovariole number were relatively high, we wanted to verify all candidate genes implicated by these variants through additional testing. Our strategy was to test every gene in our candidate list using available stock having either a *Minos* *Mi{ET1}* or *P{GT1}* insertion, or *UAS*-RNAi lines (Vienna Drosophila Stock Center, Vienna, Austria). We chose these collections as they have isogenic control lines, reducing the potential for spurious background effects; 43 candidate genes had stock available (Supplemental Material, Table S1 in File S2). We tested 12 *Minos* element *Mi{ET1}* insertion lines (Bellen *et al.* 2011) (Bloomington Drosophila Stock Center, Bloomington, IN), and two *P{GT1}* *P*-element insertion alleles (Bellen *et al.* 2004) of the *bin3* gene (*bin3*^BG01137^ and *bin3*^BG01146^) against their isogenic controls, w^1118^ and *w*^1118^; Canton-S B, respectively (Table S1 in File S2). We also tested 32 homozygous *UAS*-RNAi lines (Dietzl *et al.* 2007) (Vienna Drosophila Stock Center) (Table S1 in File S2). Two genes, *Mdr49* and *bru3*, were tested with both *Minos* element and RNAi constructs. Candidate gene expression was knocked-down in the somatic cells of the ovary using the GAL4 driver lines w{*}; P{w{+mW.hs}=GawB}*bab1*{PGal4-2}/TM6B, Tb from the Bloomington Stock Center (6803) and *TJ*-Gal4/ CyO Kr-Gal4, *UAS*-GFP (gift of B. Oliver) (Li *et al.* 2003). Male GAL4 driver flies were mated to *UAS*-RNAi females and ovariole traits of female progeny were compared to the isogenic control *y*,*w*^1118^;*P*{attP, *y*^+^, *w*^3^}. For each *P*-element, *Minos* or RNAi line, we assayed ovariole phenotypes in 10 female flies per replicate. We replicated each assay three times, for a total of 30 flies measured per line. We used the ANOVA model *Y* = *μ* + *R* + *G* + R×G + *ε*, where *R* is the fixed effect of experimental replicate, *G* is the fixed effect of genotype and *ε* is the error term, to identify genes for which there were significant differences in ovariole number between knockdown lines and isogenic controls.

The two Gal4 drivers we used could affect gene knockdown differently. To determine the influence of driver genotype on our RNAi results, we partitioned the variance in ovariole number using the ANOVA model *Y* = *μ* + *R* + *G* + *D* + *R*×*G* + *R*×*D* + *G*×*D* + *R*×*G*×*D* + *ε*, where *Y* is the difference in ovariole number between an RNAi knockdown fly and its contemporaneous isogenic control, *R* is the effect of experimental replicate, *G* is the effect of RNAi line genotype, *D* is the effect of driver genotype, and *ε* is the error term.

We observed dramatic, qualitative effects on ovary morphology when the candidate genes *CG32000* and *zfh1* were knocked down. To quantify the effects of these changes on female productivity (number of offspring), we individually mated 10 virgin female *zfh1* and *CG32000* flies to isogenic control males. Parents were cleared from culture vials after 3 d. We counted all live offspring for 5 d, beginning 11 d after initial mating. We compared offspring counts across four conditions: 10 isogenic control males individually mated to 10 isogenic control virgin females, 10 male knockdown/mutant flies individually mated to 10 virgin isogenic control females, 10 isogenic control males individually mated to 10 knockdown/mutant females, and 10 virgin knockdown/mutant males individually mated to 10 knockdown/mutant females. We repeated each assay twice, resulting in measures from 20 female flies per gene/condition. Eighteen vials in the *CG32000* assay did not produce live offspring; these failed cultures were more prevalent among vials with *CG32000* male parents, suggesting a potential and unanticipated male effect on productivity. We restricted the analysis to offspring-producing vials and used the ANOVA model *Y* = *μ* + *M* + *F* + *M*×*F* + *R*(*M*×F) + *ε*, where *Y* is the number of offspring, *M* is the fixed effect of male genotype, *F* is the fixed effect of female genotype, *R* is the random effect of experimental replicate, and *ε* is the error term. In addition, we used a PCR assay to verify the presence of the *Minos* insertion in *CG32000*. We used two amplifications, each with one primer placed within the *Minos* element, and one in *CG32000* flanking sequence. Primer pairs were 5′-GAGCCTGCGGATGAAGATC-3′ (*CG32000* flanking) and 5′-GGCGCACTTCGGTTTTTCTT-3′ (*Minos*) and 5′-GTACAATTTACAAAGGATTCGACGTGG-3′ (*CG32000* flanking) and 5′-CATTACGCCGCGTTCGAATT-3′ (*Minos*). The presence of the *P*-element was verified in both assays (Figure S1 in File S1).

To identify pleiotropic effects of ovariole number candidate genes on other ovariole phenotypes, we measured ovariole asymmetry and ovariole number *CV_E_* in all mutant and RNAi knockdown lines (Table S1 in File S2). We used the ANOVA model *Y* = *μ* + *R* + *G* + *R*×*G* + *ε* and *Y* = *µ* + *G* + *ε* for ovariole asymmetry and ovariole number *CV*_E_, respectively, where *R* is the fixed effect of experimental replicate, *G* is the fixed effect of knockdown line genotype, and *ε* is the error term, to determine whether there are significant differences in ovariole number *CV*_E_ and asymmetry between mutant or knock-down lines and their isogenic controls. We calculated the FDR for all tests (Benjamini and Hochberg 1995). All ANOVA models were evaluated using SAS software (SAS Institute).

### Pleiotropy with sleep

We identified genes common to sleep and ovariole number by comparing our ovariole number GWAS results to a previous GWAS examining 14 sleep and activity phenotypes (Harbison *et al.* 2013). In that study, sleep and activity traits were measured in 167 lines of the DGRP, which we describe briefly here. DGRP lines were divided into four blocks; each block was replicated four times, resulting in sleep and activity measurements for 32 flies/sex/line. Virgin males and females were loaded into Trikinetics (Waltham, MA) activity monitors. Sleep was assayed, and sleep phenotypes were calculated as detailed below (*Sleep phenotypes*). All DGRP variants with a minor allele frequency ≥0.0238 were tested for association with sleep traits using two models: (1) a GLM model similar to the one described above except that the effect of sex was incorporated as an additional factor; and (2) a FaST-LMM model incorporating population structure. Day average bout length in males was the only phenotype adjusted for *Wolbachia* infection status, as this was the only phenotype affected. In addition, some sleep characteristics were associated with chromosomal inversions (Harbison *et al.* 2013). Variants were called significant if their FDR ≤0.01 in the original study, but, for the purposes of comparison with the GWA described here, we used the same significance level for sleep as for ovariole number traits—a *P*-value of 1 × 10^−5^.

We defined a gene as pleiotropic if it contained one or more polymorphic variants significantly associated with ovariole number, and one or more variants significantly associated with at least one sleep trait. For both groups of traits, we considered both variants that map to gene coding sequences, and those within a 1 kb window up or downstream of the coding sequence.

### Sleep phenotypes

We tested the effect on sleep of 20 candidate pleiotropic ovariole number/sleep genes using mutant alleles and RNAi constructs. These genes were identified through the GWAS comparison using a 1 × 10^−5^ *P*-value threshold. In addition, mutant alleles of *bin3* were previously shown to affect day and night sleep duration in a study of genome-wide transcriptional abundance (Harbison *et al.* 2009). We tested these same alleles in this study for both sleep and ovariole number traits. All genes were tested either with mutations or RNAi-induced knockdown except *bru3*, which was tested with both (Table S1 in File S2). For sleep tests using *UAS*-RNAi constructs, we used the Gal4 driver line *P*{{*w*^+^Mc} = Gal4-*elav*.L}2/*CyO* (8765) from the Bloomington Stock Center to reduce candidate gene expression in all neurons (Harbison *et al.* 2013).

We recorded 5 d of continuous sleep activity from 16 male and 16 female flies per genotype using the *Drosophila* Activity Monitoring System (Trikinetics) as in (Harbison *et al.* 2013). Trikinetics monitors record the activity of each individual fly by counting the number of times a fly breaks an infrared beam each minute. Prior to sleep assays, virgin flies collected from each line were held at 20 flies per same-sex vial to control for the effects of mating (Isaac *et al.* 2010) and social exposure (Ganguly-Fitzgerald *et al.* 2006) on sleep. Data from flies that did not live through the assay were removed prior to analysis. A C# program (R. Sean Barnes, personal communication) was used to calculate 16 sleep measures from the raw activity data. Sleep parameters included the seven mean and seven *CV_E_* traits previously measured (Harbison *et al.* 2013), plus two additional traits, *i.e.*, sleep latency and its coefficient of environmental variation. Sleep is defined as five or more continuous minutes of inactivity (Huber *et al.* 2004). Using this definition, we calculated the amount of time the flies spent sleeping during the day or night; the number of sleep bouts during the day or night; the average length of a sleep bout during the day or night; and the number of activity counts per minute spent awake (waking activity). We defined sleep latency as the number of minutes from the start of the night until a fly’s first sleep bout. The coefficient of environmental variation (*CV*_E_) was calculated for these traits as well. Sleep measurements were replicated three times, resulting in measurements from 48 flies per sex per line. We analyzed these data for both sexes combined using the ANOVA model *Y* = *μ* + *S* + *G* + *S*×*G* + *R*(*S*×*G*) + *ε*, where *S* is the fixed effect of sex, *G* is the fixed effect of genotype, *R*(*S*×*G*) is the random effect of experimental replicate on the interaction of sex and genotype, and *ε* is the error term. Sleep and its genetic basis differ between male and female flies (Hendricks *et al.* 2003; Huber *et al.* 2004; Isaac *et al.* 2010; Harbison *et al.* 2013; Guo *et al.* 2016), which we examined with the reduced model *Y* = *μ* + *R* + *G* + *R*×G + *ε*, where *G* is the fixed effect of genotype and *ε* is the error term. For *CV_E_* traits, we used the model *Y* = *μ* + *S* + *G* + *S*×*G* + *ε*, where *S* is the fixed effect of sex, *G* is the fixed effect of genotype, and *ε* is the error term. Reduced models for *CV*_E_ traits were computed as *Y* = *µ* + *G* + *ε* for each sex separately. All ANOVA models were evaluated using SAS software (SAS Institute).

### Data availability

All data necessary to replicate our analyses are available as supporting files except for raw ovariole trait counts for each fly, which are available upon request. Table S2 in File S2 lists ovariole number, ovariole asymmetry, ovariole number *CV*_E_, and ovariole asymmetry *CV*_E_ mean phenotypes for each DGRP line tested; Table S5 in File S2 lists these means adjusted for the effects of *Wolbachia* infection and chromosome inversion.

## Results

### Quantitative genetic analyses

**Figure 1:**
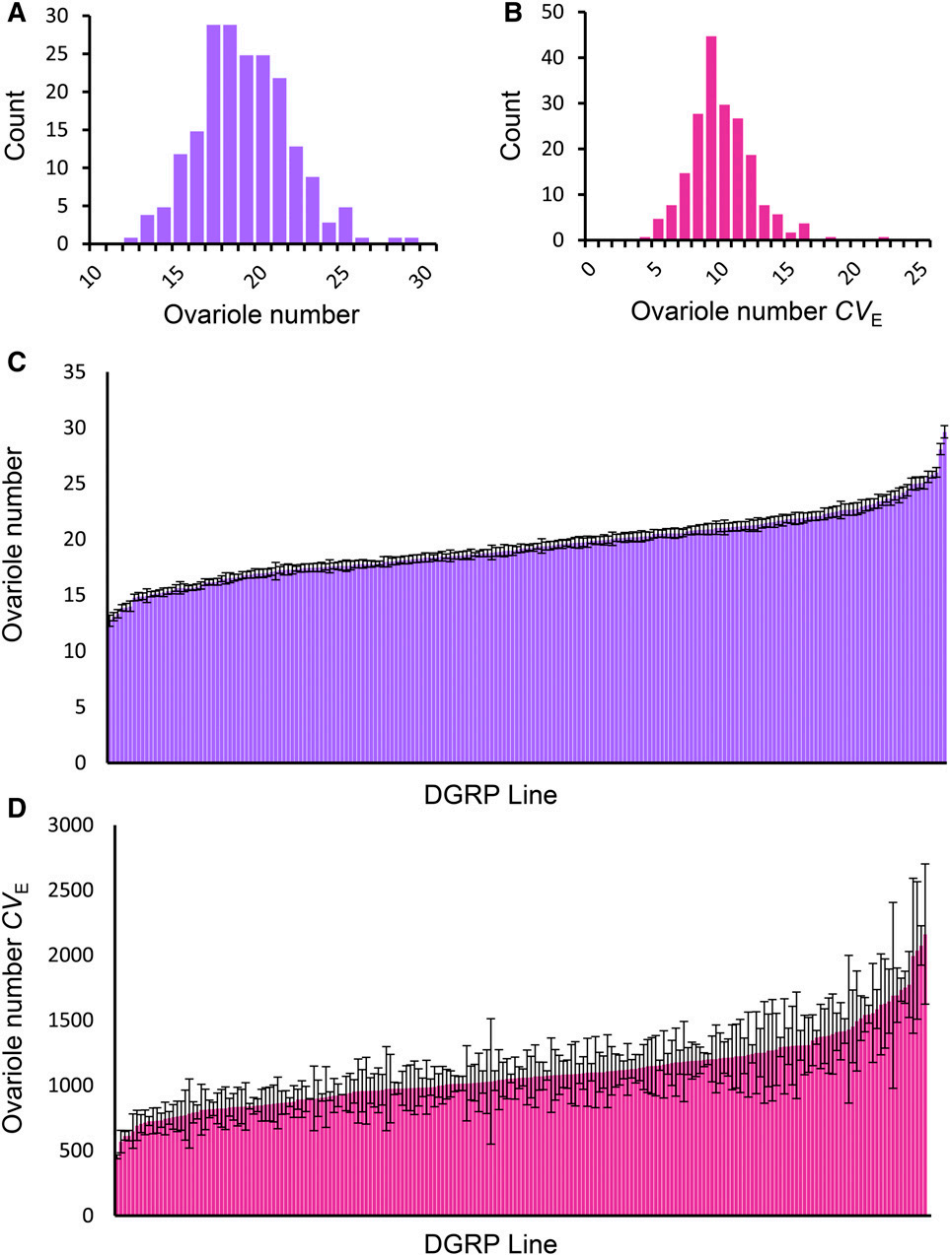
Variation in ovariole number in the DGRP. Histogram of (A) ovariole number and (B) ovariole number *CV*_E_ line means. Mean and SE of (C) ovariole number and (D) ovariole number *CV_E_*.

**Figure 2:**
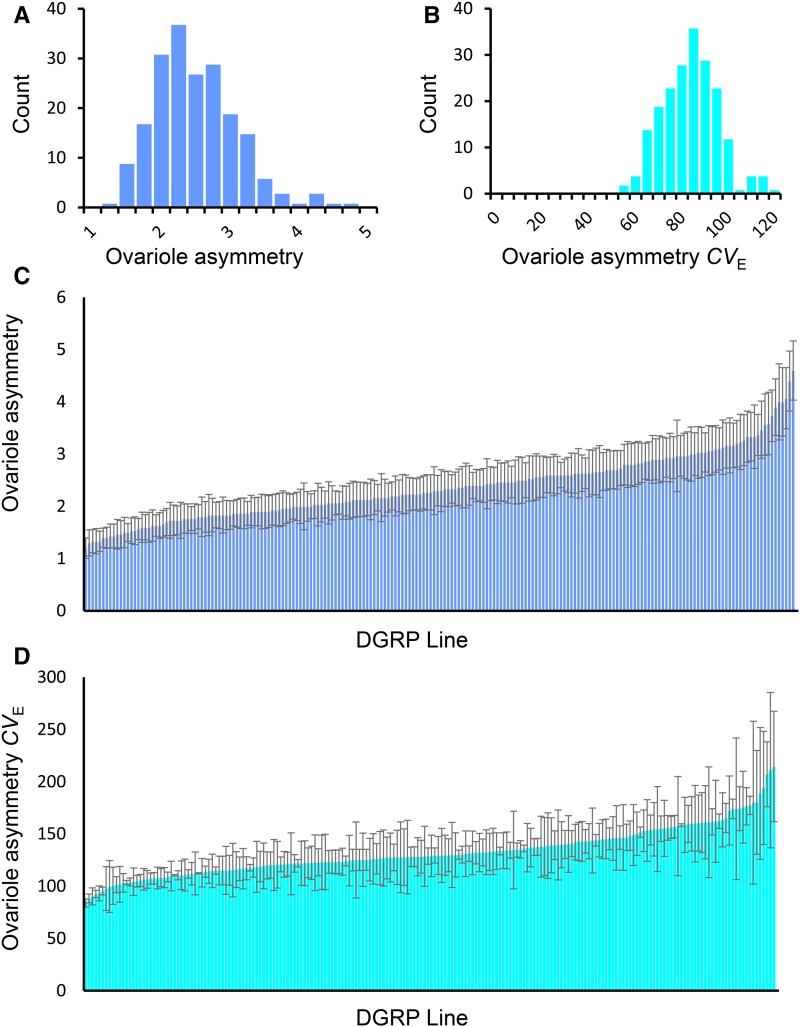
Variation in ovariole asymmetry in the DGRP. Histogram of (A) ovariole asymmetry and (B) ovariole asymmetry *CV*_E_ line means. Mean and SE of (C) ovariole asymmetry and (D) ovariole asymmetry *CV_E_*.

**Figure 3:**
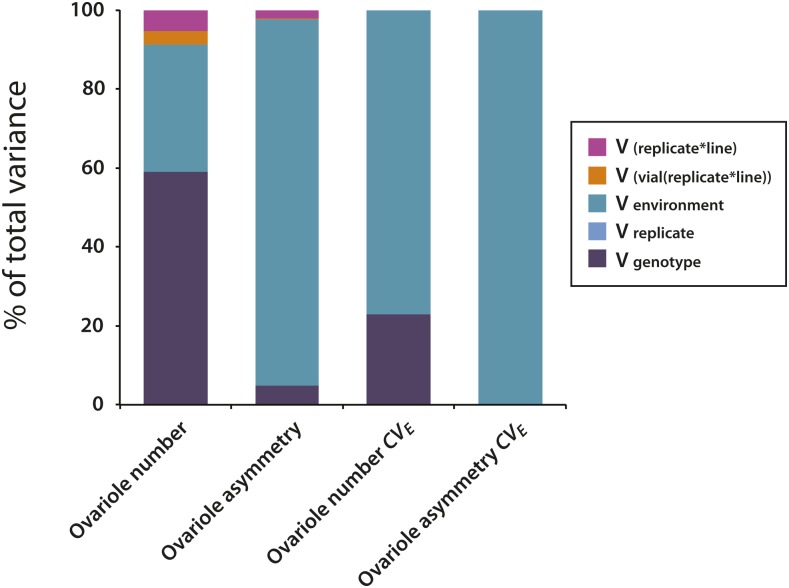
Partitioning of variance components for ovariole phenotypes.

The mean values of both ovariole number and right/left ovariole asymmetry varied substantially among DGRP lines, ranging from 12.7 to 29.6, and from 1.2 to 4.6, respectively (Figure 1 and Figure 2 and Table S2 in File S2). Quantitative genetic analyses uncovered a highly significant genetic variance component in both traits (*P* < 0.0001) (Figure 3 and Table S3 in File S2), with a broad-sense heritability (*H*^2^) of 0.59 estimated for ovariole number, and a *H*^2^ of 0.05 estimated for ovariole asymmetry, consistent with previous estimates (Robertson 1957; Wayne *et al.* 2001). Thus ovariole number is strongly influenced by genotype, while ovariole asymmetry is dominated by environmental factors. To determine whether common genes might affect ovariole number and asymmetry, we computed the genetic correlation between the traits. We found that ovariole number and asymmetry were genetically correlated (*r*_G_ = 0.72), which suggests that a proportion of the genes affecting each trait are shared between them (Table S4 in File S2). Thus, to the limited extent that ovariole asymmetry is determined by genetics, it may share a common genetic and developmental architecture with ovariole number.

The coefficient of environmental variation (*CV_E_*) indicates the sensitivity of quantitative traits to the environment (Mackay and Lyman 2005), with higher *CV_E_* values reflecting greater variability in the trait due to random environmental perturbations. In this study, ovariole number *CV_E_* ranged from 4.9 to 22.1, while the *CV_E_* of ovariole asymmetry ranged from 50.3 to 119.6 (Figure 1 and Figure 2 and Table S2 in File S2). The sensitivity of a trait to the environment can be influenced by both genes and the environment, with the genetic component reflecting the degree of canalization (Hill and Mulder 2010). To determine the relative contributions of genetic and environmental factors to ovariole number and ovariole asymmetry *CV_E_*, we partitioned the traits’ variance into their genetic and environmental components. We observed a highly significant effect of genotype (*P* < 0.0001, *H*^2^ = 0.23) in ovariole number *CV_E_*, but could not detect a genetic component of variance in ovariole asymmetry *CV_E_* (*H*^2^ = 0; *P* = 0.65) (Figure 2 and Table S3 in File S2). Thus, genetic factors can influence differences in ovariole number among individual flies of a given genotype, but the degree of asymmetry in ovariole number among individual flies is dominated by environmental or stochastic fluctuations.

We calculated the genetic correlation between ovariole number and ovariole number *CV_E_*_._ We found a weak negative correlation among the traits (*r*_G_ = −0.16), indicating little overlap in the genes affecting them. Last, we found that ovariole number *CV_E_* and the mean value of ovariole asymmetry were highly correlated (*r*_G_ = 0.70) (Table S4 in File S2). We anticipated this because both traits reflect the organisms’ response to environmental “noise” and thus they may share a common genetic basis. Given the highly significant genotypic contribution to ovariole number, its coefficient of variation and asymmetry we expected to identify genetic variants underlying these traits with some variants in common and others unique to each trait.

### Genotype-phenotype associations

We used three approaches to examine potential genotype-phenotype associations for ovariole number, ovariole asymmetry, and ovariole number *CV*_E_. In each analysis, we considered all variants occurring at a frequency of 1% or greater in the DGRP. The most basic approach was a simple GLM model implemented using SAS. This model does not require the imputation of missing variant allele calls, and did not account for *Wolbachia* infection status, presence/absence of chromosomal inversions, or population structure, known confounders of association studies in the DGRP (Huang *et al.* 2014). The other two approaches used FaST-LMM to calculate genotype–phenotype associations (Lippert *et al.* 2011); to obtain increases in computational speed, FaST-LMM uses mean imputation to call alleles for missing variants. Both FaST-LMM analyses used ovariole phenotypes corrected for the effects of *Wolbachia* infection and chromosomal inversions. One FaST-LMM analysis accounted for population structure, while the other did not. We observed little effect of population structure on these analyses, and ovariole number traits were only mildly affected by *Wolbachia* infection and chromosomal inversions, though they may have impacted ovariole number *CV*_E_. Thus, we observed a high level of concordance among all three analyses. We present the combined results of the three analyses here, and address differences among them in the *Discussion*.

Quantile–quantile (QQ) plots constructed from the GWA results revealed appreciable deviation of observed from expected *P*-values for each trait (Figures S2–S4 in File S1). These deviations may reflect population structure, LD, or epistasis. One of our analyses corrected for population structure by incorporating the relationship matrix into the mixed-model analysis (Lippert *et al.* 2011); this analysis resulted in some slight improvement in the QQ plot or *P*-value distributions, suggesting little effect of population structure on the results. Our use of polymorphic variants with a minor allele frequency of <5% increases the potential of spurious associations due to long-range LD (Huang *et al.* 2014). Accordingly, we calculated the extent of LD among significant variants. We observed high LD (*r*^2^ ≥ 0.80) in a small region on chromosome 2R (base-pair position 8583734–5854540), a small region on chromosome *3R* (base-pair position 22162040–22178605), and virtually all of the significant polymorphisms on chromosome 4 (Figure S5A). Ovariole asymmetry showed little LD (Figure S5B), while ovariole *CV*_E_ had a region of LD on chromosome 3R (base-pair position 12970391–13034018) (Figure S5C). These high-LD regions make causal polymorphisms more difficult to distinguish, despite the fact that LD decays within 10–30 bp on average in the DGRP (Mackay *et al.* 2012). In addition, adjustment of phenotypes for the effect of five common inversions and for *Wolbachia* infection status before GWA analysis did not impact ovariole number or ovariole asymmetry. The adjustment may have affected ovariole number *CV*_E_, though the correlation between the adjusted and unadjusted trait values was very high (0.968) (Figure S4B in File S1; cf. Table S2 in File S2 and Table S5 in File S2). However, none of the inversions nor infection status were significantly associated with any of the three ovariole phenotypes (Table S6 in File S2). We hypothesized that variants identified by this study would causally contribute to quantitative variation in ovariole phenotypes, with the caveat that causal polymorphisms would be more difficult to pinpoint in regions of high LD.

We limited our significance threshold to an uncorrected *P*-value of ≤ 1 × 10^−5^, a threshold applied in many quantitative trait studies in the DGRP (Arya *et al.* 2015; Dembeck *et al.* 2015a,b; Garlapow *et al.* 2015; Shorter *et al.* 2015; Zwarts *et al.* 2015; Hunter *et al.* 2016). Using this threshold, we identified 164 variants associated with ovariole number with FDRs ranging from 0.057 to 0.430 (Table S7 in File S2). For ovariole number asymmetry, 175 variants were associated with FDRs ranging from 0.043 to 0.293 (Table S8 in File S2). In addition, 1855 variants were associated with ovariole number *CV_E_*, and FDRs ranged from 0.0002 to 0.042 (Table S9 in File S2). We were primarily interested in ovariole number, so we focused on the variants associated with it. However, many of the variants for ovariole number had high FDRs, which indicated less confidence in these variant calls. We addressed this issue with further analysis and testing (see below).

Significantly associated SNPs covered the complete allele frequency spectrum, with MAFs ranging from 0.01 to 0.49. Low frequency alleles (MAF <0.05) were associated with each trait, and had larger normalized effect sizes than the more common alleles (Figure S6 in File S1). Consistent with the results of numerous GWA studies (Maurano *et al.* 2012), the majority (88%) of significant trait-associated variants mapped to noncoding regions of the genome. Many of these (43%) were in introns (Figure S7 in File S1). In contrast, just 12% of identified variants were in gene coding regions. Thus, polymorphisms associated with ovariole number and its related traits tend to fall in noncoding regions, and large-effect variants tend to be rare.

Some of the variants were predicted to alter amino acids. Nonsynonymous variants in seven genes (*beat-IIb*, *bin3*, *CG32006*, *CG33978*, *CG34427*, *Ir67b*, and *Rad23*) and seven genes (*CG10621*, *CG10623*, *CG11149*, *CG2023*, *Plap*, *sip1*, and *Tsf1*) were associated with ovariole number and ovariole number asymmetry, respectively. There were 135 nonsynonymous variants associated with ovariole number *CV_E_*. The amino-acid altering variants associated with ovariole number *CV_E_* mapped to 86 unique genes. Notably, 16 of them were in *CG14117*, a gene of unknown function. Although they were a minority of all variants associated with ovariole traits, we would expect many of these nonsynonymous substitutions, insertions and deletions to significantly affect *Drosophila* female reproductive morphology.

To assess pleiotropy among ovariole traits, we investigated the degree to which ovariole number, asymmetry, and ovariole number *CV_E_* shared common genetic architectures. No variants were common to all three traits and among all possible two-way unions. While pleiotropy among polymorphisms was nearly nonexistent, we identified 27 genes common to multiple ovariole traits (Table S10 in File S2).

Several of the genes we found associated with ovariole number, asymmetry, and ovariole number *CV_E_* were biologically plausible candidates for affecting ovariole development. These included genes with known function in *Drosophila* sexual differentiation (*fru*) (Ryner *et al.* 1996), germ cell migration (*Mdr49*) (Ricardo and Lehmann 2009), and oogenesis (*zfh1*, *cher*, and *bin3*) (Moore *et al.* 1998; Sokol and Cooley 2003; Singh *et al.* 2011). While experimental work is needed to confirm causal alleles, these genes are candidate determinants of *Drosophila* fitness.

### Functional verification of ovariole candidate genes

We used all available *Minos* (Bellen *et al.* 2011) and *P*-element insertion lines (Bellen *et al.* 2004), and homozygous *UAS*-RNAi constructs (Dietzl *et al.* 2007) to evaluate candidate genes for ovariole number. Of the 89 genes identified by the GWA, 46 constructs in 43 candidate genes were available (Table S1 in File S2). These were genes in which SNPs located within or up to 1000 bp up or downstream of the gene coding sequence were significantly associated with ovariole number in our GWA analyses. Tests of *Minos* and *P*-element lines revealed significant effects of nine genes (Table 1 and Table S11 in File S2). Notably, we observed a reduction in ovariole number in all mutant lines except MB^00990^, which carries a *Minos* element in *kirre*. In tests using *UAS*-RNAi lines, we reduced candidate gene expression in somatic cells of the ovary using the Gal4 driver lines *bab1*-Gal4 and *traffic jam*-Gal4 (see *Materials and Methods*). The transcription factor *bric-a-brac 1* (*bab1*) is expressed during the larval stage by terminal filament precursor, and terminal filament cells of the ovary (Couderc *et al.* 2002). Loss of function of *bab1* has been shown to affect ovariole number by altering terminal filament cell proliferation (Bartoletti *et al.* 2012). *traffic jam* (*tj*) is a large Maf transcription factor expressed by somatic gonadal cells throughout ovarian development. It affects terminal filament morphogenesis by regulating the expression of adhesion molecules as the ovary develops (Li *et al.* 2003). Thus, these two Gal4 drivers may identify genes that affect ovariole number through different cell populations and developmental mechanisms.

**Table 1:** Candidate genes with significant effects on ovariole number

Construct Type	Gene	*P*-Value	Ovariole Number Difference	MAF(s)
*P*-element	***bin3****^a^*^,^*^b^*^,^*^c^*^,^*^d^*	**0.0024**	**−1.51**	**0.359**
*Minos element*	***Ank****^a^*	**<0.0001**	**−4.60**	**0.085**
	***bru3****^a^*^,^*^b^*^,^*^c^*^,^*^e^*	**<0.0001**	**−3.58**	**0.015**
	***CG30288****^a^*^,^*^b^*^,^*^c^*	**<0.0001**	**−2.52**	**0.051, 0.056**
	***CG42389****^a^*^,^*^b^*^,^*^c^*	**<0.0001**	**−4.52**	**0.027**
	***kirre****^b^*	**<0.0001**	**3.02**	**0.449**
	**Lip4***^a^*^,^*^b^*	**0.0004**	**−2.37**	**0.286**
	***Mdr49****^a^*^,^*^b^*^,^*^c^*^,^*^e^*	**0.0001**	**−2.17**	**0.010**
	***VAChT****^c^*	**0.0080**	**−1.48**	**0.479**
*bab1-Gal4/UAS-RNAi*	***CG31999****^a^*^,^*^e^*	**<0.0001**	**−3.38**	**0.069, 0.087, 0.098**
	***RhoGEF64C****^a^*^,^*^b^*^,^*^c^*^,^*^e^*	**0.0064**	**−1.69**	**0.010, 0.065**
	***Mdr49****^a^*^,^*^b^*^,^*^c^*^,^*^e^*	**0.0001**	**−2.76**	**0.010**
	***SdhA****^b^*^,^*^e^*	0.0350	−1.44	0.060, 0.061
*tj-Gal4/UAS-RNAi*	*blot**^a^*	0.0243	−0.95	0.213
	***bru3****^a^*^,^*^b^*^,^*^c^*^,^*^e^*	**<0.0001**	**−2.35**	**0.015**
	***CG10494****^a^*^,^*^b^*^,^*^c^*	**0.0006**	**−2.43**	**0.051, 0.056**
	*CG1674**^a^*	0.0440	−1.56	0.080
	**Arl4(*CG2219*)***^a^*	**0.0023**	**−1.70**	**0.082**
	***CG31999****^a^*^,^*^e^*	**<0.0001**	**−2.89**	**0.069, 0.087, 0.098**
	*CG34408**^a^*^,^*^b^*	0.0277	−1.36	0.434, 0.443
	***Crk****^a^*	**0.0121**	**−1.38**	**0.083, 0.089**
	*fred**^b^*^,^*^c^*	0.0206	−1.28	0.100, 0.109
	***RhoGEF64C****^a^*^,^*^b^*^,^*^c^*^,^*^e^*	0.0490	−1.29	0.01, 0.065
	***Mdr49****^a^*^,^*^b^*^,^*^c^*^,^*^e^*	**0.0025**	**−1.95**	**0.010**
	***Mst57Dc****^a^*^,^*^b^*^,^*^c^*	**0.0019**	**−2.00**	**0.010**
	*Nlg1**^a^*^,^*^b^*^,^*^c^*	0.0432	−1.18	0.023
	***SdhA****^b^*^,^*^e^*	**<0.0001**	**−2.45**	**0.060, 0.061**
	***slim****^b^*	**0.0016**	**−1.82**	**0.128**

*P*-values reflect the combined analysis of three experimental replicates. Ovariole number difference is the difference in mean ovariole number between mutant or knockdown lines and their isogenic controls. MAF, minor allele frequency. Genes with FDR ≤0.05 indicated in bold type; genes without bolding have FDR < 0.15. Note that *anneboleyn* (*CG32000*)*^a^* and *zfh1^a^*^,^*^b^*^,^*^c^* had major effects on ovary morphology and are not listed here.

a Phenotypes corrected for *Wolbachia*, chromosomal inversions; associations incorporate population structure.

b Phenotypes corrected for *Wolbachia*, chromosomal inversions; associations do not incorporate population structure.

c Phenotypes not corrected for *Wolbachia* or chromosomal inversions; associations do not incorporate population structure.

d Allele *bin3*^BG01137^ only.

e Genes significant in multiple assays.

Tests of RNAi constructs showed significant phenotypic effects of gene knockdowns for 15 genes (Table 1). Interestingly, the efficacy of gene expression knockdown was different with *bab1* or *traffic jam* drivers (Table S12 in File S2). Four RNAi constructs in which gene expression was reduced using the *bab1* driver showed phenotypic effects, *vs.* 15 constructs driven by *traffic jam*. Notably, different genes impacted ovarioles with the *bab1* and *tj* drivers, though *CG31999*, *Mdr49*, *RhoGEF64C*, and *SdhA* had significant ovariole differences from the control using both drivers. Thus, the effects of individual genes on ovariole number are mediated by the cellular localization and developmental timing of their expression.

**Figure 4:**
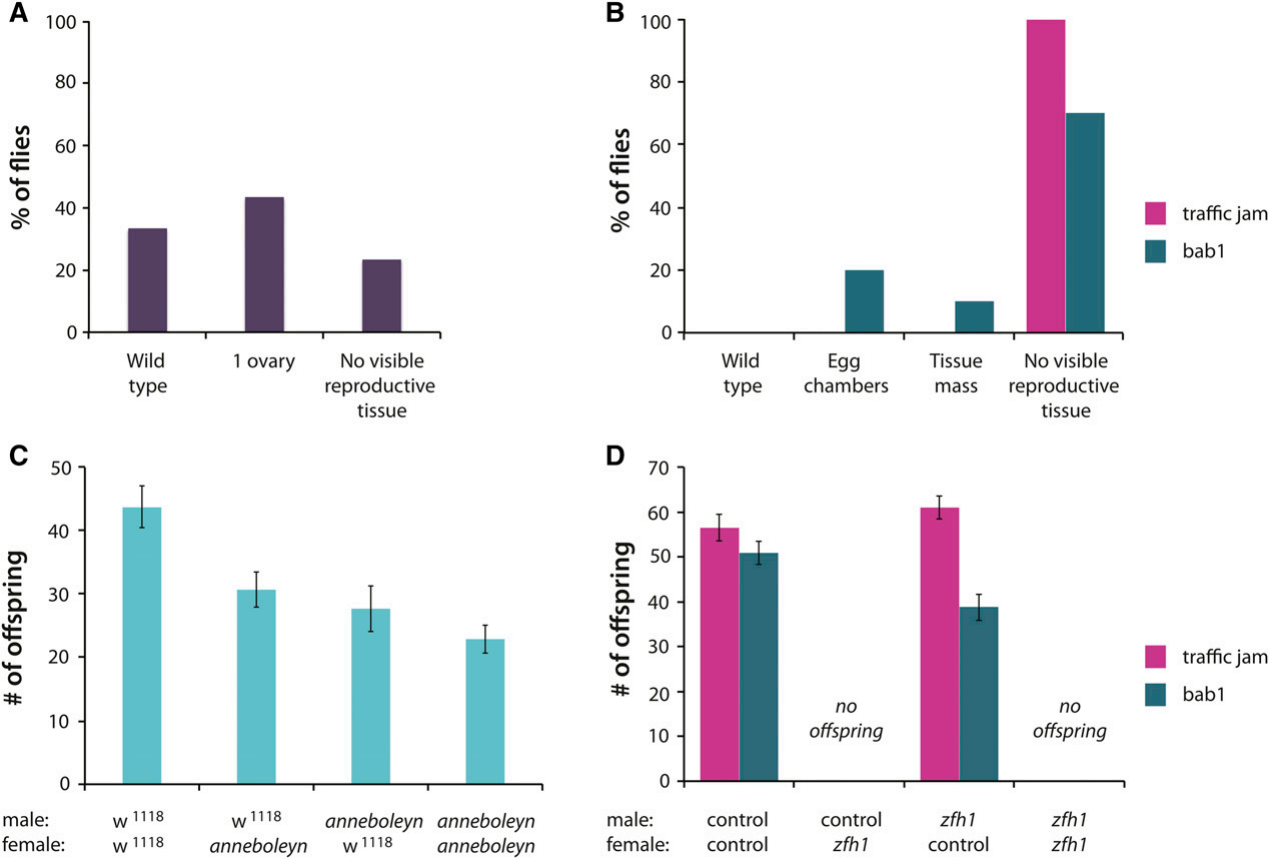
Impact of major effect genes on ovary morphology and female fecundity. (A) *anneboleyn* mutant, and (B) *zfh1* knockdown ovary morphology; (C) *anneboleyn* mutant, and (D) *zfh1* knockdown offspring production.

In addition to these quantitative changes in ovariole number, we observed qualitative disruptions to reproductive morphology in flies carrying a *Minos* element in *CG32000* and RNAi-mediated knockdown of *zfh1*. Of *CG32000* females, 66% had either just one ovary or no visible reproductive tissue; the remaining flies examined had ovaries that were wild type in appearance (Figure 4A). We also observed large-scale disruptions to ovary morphology in all females with an RNAi construct in *zfh1*. The putative reduction of *zfh1* resulted in no visible reproductive tissue in 100% (*tj-Gal4* driver) and 70% (*bab1-Gal4* driver) of flies tested. The remaining *bab1* flies had large-scale reproductive abnormalities: either a single, large, mass of tissue in place of ovaries, or free-floating egg chambers without a visible ovary (Figure 4B). To determine the effects of these morphological abnormalities on female reproductive fitness, we measured the productivity of individual females by counting the number of offspring they produced. Productivity in *CG32000* females was reduced as compared to the isogenic control (*P* = 0.0269; Figure 4C). While crosses to *CG32000* males also had reduced progeny relative to crosses with isogenic control males, the effects were not significant (*P* = 0.7774). There was a highly significant effect of *zfh1* knockdown on fecundity when we used both the *bab1* (*P* = 0.0014) and *traffic jam* (*P* < 0.0001) (Figure 4D) Gal4 drivers. All females carrying the *zfh1* RNAi construct were sterile. Reducing *zfh1* expression in males with the *bab1* driver decreased productivity relative to control males (*P* < 0.05) (Figure 4D). *zfh1* has a well-known role in *Drosophila* gonad development (Moore *et al.* 1998), but the function of *CG32000* in ovariole development is unknown. These tests confirm the critical role of *zfh1* in female fitness and identify *CG32000* as a novel gene affecting female reproductive morphology and fitness. Due to the reduction in female productivity, we have renamed *CG32000 anneboleyn* (*anne*), after King Henry VIII of England’s wife Anne Boleyn, who had fertility issues.

Although we chose these genes as candidates affecting ovariole number, we also observed significant effects on ovariole asymmetry (Table S13 in File S2). Seven genes (*bin3*, *blot*, *CG31999*, *CG33970*, *Ziz*, *SdhA*, and *slim*) also impacted ovariole asymmetry in these tests. These effects suggested a shared genetic architecture underlying ovariole number and the processes that control its variation within an individual, though additional replication would be required to confirm these effects as ovariole asymmetry is dominated by environmental factors. Interestingly, none of the genes impacting ovariole number were also observed to affect ovariole number *CV_E_* (Table S13 in File S2). This may be attributed to the difficulty in obtaining accurate estimates of *CV*_E_ (Mulder *et al.* 2007), or to differences in the genetic bases of these traits.

### Pleiotropy with sleep

We computed genetic correlations between ovariole phenotypes and female values for 14 sleep/activity traits measured in the DGRP (Harbison *et al.* 2013). We found that ovariole number and asymmetry were essentially uncorrelated with all sleep/activity traits. In contrast, there were stronger genetic correlations between ovariole number *CV_E_* and some sleep/activity *CV_E_* traits (*r*_G_ = 0.40 for night bout number *CV_E_*, and *r*_G_ = 0.37 for waking activity *CV_E_*) (Table S14 in File S2). This suggests common variants affecting environmental sensitivity in both phenotypes exist.

Although ovariole number and sleep traits showed little genetic correlation, we noted that 39 genes contained variants significantly associated both with ovariole number and one or more of the 14 measured sleep traits (Harbison *et al.* 2013). Pleiotropy has been observed previously where individual SNPs in a given gene did not overlap among traits, but were individually associated with each trait (Carbone *et al.* 2006). Interestingly, over half of these candidate genes were associated with >1 sleep trait (Tables S15 and S16 in File S2). In addition, 42 ovariole asymmetry and 276 ovariole number *CV*_E_ genes overlapped with sleep traits (Table S15 in File S2). With both sets of traits, we examined the overlap among genes with variants having nominal *P*-values of 1 × 10^−5^ or less. However, the number of genes overlapping among any two data sets tends to scale linearly with the number of genes identified (S. Harbison, unpublished data). Thus, some overlap between sleep and ovariole number candidate genes existed, but to demonstrate pleiotropy, functional tests of candidate genes for their role in sleep were necessary.

We tested 20 of the mutations and gene knockdowns that we assessed for ovariole number for pleiotropic effects on sleep. Some effects on sleep were sex-specific (Table S17 in File S2), while others were common to both males and females (Table S18 in File S2). These tests verified multiple GWAS findings of association between individual genes and specific sleep phenotypes (Harbison *et al.* 2013). Three, seven, and eight GWAS predictions were verified for females, males, and both sexes, respectively. In addition, we replicated the effects of *bin3* mutations on both day and night sleep duration (Tables S17 and S18 in File S2) (Harbison *et al.* 2009). Nearly every gene affected >1 sleep trait, which is consistent with the extensive pleiotropy among sleep candidate genes that was previously observed (Harbison *et al.* 2013). Importantly, these tests identified seven genes with verified functional effects on both sleep and ovariole number: *bin3*, *blot*, *CG42389*, *kirre*, *slim*, *VAChT*, and *zfh1* (Table S19 in File S2). These genes may have pleiotropic effects on both sleep and ovariole number; alternatively, the apparent commonality may be due to linkage among candidate polymorphisms (Hughes and Leips 2017). Thus, we calculated the linkage disequilibrium among 269 candidate polymorphisms in the 20 genes common to sleep and ovariole number that were tested. Only three pairs of polymorphisms between ovariole number and sleep had *r*^2^ values that were significantly different from zero. Within the gene *VAChT*, *r*^2^ was 0.417 between base-pair positions 18707150 and 18711650 on chromosome *3R* for ovariole number and night average bout length, respectively. Two day bout number *CV*_E_ polymorphisms (base-pair positions 16631610 and 16635912 on chromosome *2L*) in *CG42389* had high *r*^2^ values (0.333 and 1.0, respectively) with ovariole number polymorphism 16608818. Thus, we identified seven genes that exhibited pleiotropic effects on sleep and ovariole number, but the extent of LD among candidate SNPs within these genes was low.

## Discussion

We used the DGRP to identify polymorphisms underlying natural variation in ovariole number, ovariole *CV*_E_, and ovariole number asymmetry. Only a few genes underlying ovariole number were previously known. These are the insulin-like receptor *InR*, the transcription factor *bab1*, and members of the *Hippo* pathway (*hpo*, *yki*, *sav*, *Mer*, and *ex*) (Green and Extavour 2012, 2014; Sarikaya *et al.* 2012; Sarikaya and Extavour 2015). We identified 164 unique polymorphisms in 89 genes implicated in ovariole number variation in this study. Consistent with the genetic architecture of other complex traits (Jordan *et al.* 2012; Mackay *et al.* 2012; Weber *et al.* 2012; Harbison *et al.* 2013), the majority of polymorphisms affecting variation in ovariole number were at low frequency. These variants, which segregate in the DGRP, have passed through natural selection’s filter. Given ovariole number’s importance to reproductive fitness, we anticipate that some of its genetic determinants will have been fixed by natural selection in the DGRP. For instance, mean ovariole number differs tremendously among insects and also varies among *Drosophila* species (Hodin and Riddiford 2000; Hodin 2009). This suggests the existence of fixed variants that determine upper and lower limits for ovariole number in a species. These cannot be detected by GWAS but likely still are important to reproductive physiology.

Most of the variants we reported for ovariole number traits would have been discovered using any of the three analysis approaches outlined here. However, the analysis that included population structure as a covariate and adjusted phenotypes for the presence of *Wolbachia* infection and chromosomal inversions did identify more candidate genes that were verified in candidate gene tests, as inspection of Table 1 reveals. Each approach yielded relatively high FDRs associated with ovariole number variants (0.057–0.43), suggesting that some of our GWAS results may be false positives. Yet many of the variants implicated to impact ovariole number lie in genes involved in female reproductive development. First, our analyses identified an intronic polymorphism in *Zn finger homeodomain 1 (zfh1)* as associated with ovariole number. *zfh1* is a component of the STAT signaling pathway essential for self-renewal of ovarian germline stem cells (Leatherman and DiNardo 2008). High expression levels of *zfh1* are needed for germ cell differentiation, and, consequently the formation of functional ovaries (Maimon *et al.* 2014). Our RNAi-mediated gene knockdown experiments in *bab1* and *tj*-expressing cells verify the necessity of *zfh1* for ovary development and female fertility. These findings mirror those of a previous study (Maimon *et al.* 2014), which also used RNAi to reduce *zfh1* expression in the ovary with a *traffic jam* Gal4 driver.

Second, eight additional candidate genes have been previously implicated in ovary development: *hts* (Snapp *et al.* 2004; Pokrywka *et al.* 2014), *cher* (Sokol and Cooley 2003), *Mdr49* (Ricardo and Lehmann 2009), *tai* (Bai *et al.* 2000), *pan* (Jordan *et al.* 2000), *RhoGEF64c* (Wang *et al.* 2006), *Nlg1*, and *VAChT* (Soshnev *et al.* 2012). The overlap with genes implicated in ovary development provides additional confidence in the GWAS predictions for these genes. Using mutant and knockout lines, we verified effects on ovariole number of four of these genes: *Mdr49*, *VAChT*, *RhoGEF64C*, and *Nlg1*. The canonical sex determination gene *fruitless* (*fru*) and *dpr13*, a *fru* target (Vernes 2014) also are candidate ovariole number genes, as is the *doublesex* and Mab-related transcription factor *dmrt93b*. Interestingly, these sex-determination genes are primarily expressed in, and associated with, the development of male reproductive morphology and behavior (Ito *et al.* 1996; Ryner *et al.* 1996; O’Day 2010). Our findings suggest a role in female reproductive development as well.

We also observed effects on ovariole number of 19 additional candidate genes, including major disruptions in reproductive morphology in *anneboleyn* mutant lines. *anneboleyn* lies within the high-LD region of chromosome 4, along with *Ank*, *CG1674*, *Arl4*, *CG31999*, *CG33978*, *Crk*, and *pan*. *Ank*, *CG1674*, *Arl4*, *CG31999*, and *Crk* all had significant quantitative effects on ovariole number, with a reduction of ovariole number with reduced gene product in each case (Table 1). We cannot rule out the possibility that there is a single causal locus in this region because of the strong LD. However, it is unlikely that the effects of the verification tests are positional, as we used *UAS*-RNAi constructs that map to a single location on the second chromosome for tests of *CG1674*, *Arl4*, *CG31999*, and *Crk* (Dietzl *et al.* 2007). *anneboleyn* is predicted by electronic annotation to function as a cell membrane protein involved in cation transport (Attrill *et al.* 2016), and is a novel reproductive gene with no previously known function. Thus, the GWAS identified genes that not only impact ovariole number, but also have major effects on ovariole morphology.

Several limitations exist when using mutations to verify candidate genes discovered using GWA. First, gene mutants and expression knockdowns may differ in their phenotypic effects from naturally segregating variants, and positional effects of engineered mutations can cause opposing phenotypes (Rollmann *et al.* 2006). Second, although the use of mutant/RNAi collections with isogenic controls greatly reduces background effects, mutations can accumulate randomly in both mutant and control lines over time, potentially resulting in off-target effects on the phenotypes of interest. Third, GWAS does not provide any information concerning the tissues or developmental stages in which candidate gene action is critical to the trait of interest. While we anticipate that the effects of natural variants may be subtle, expression changes in natural populations of *Drosophila* can vary greatly in magnitude (Ayroles *et al.* 2009). Furthermore, the expression levels of some tested genes may be reduced more significantly than others as constructs differ in their efficiency. In addition, *Minos* and *P*-element mutations impact gene expression in all cell types throughout development. The ovariole number differences we observed between RNAi lines crossed to *bab1* *vs.* *tj* Gal4 drivers demonstrates that cell type and developmental timing are important considerations in the phenotypic effects of candidate genes. Finally, the genetic backgrounds of flies having mutations or RNAi constructs differ from the DGRP lines. Thus, we suggest that epistatic interactions may modify candidate gene effects on ovariole number in the DGRP, and, in these tests, as epistasis is commonly observed among *Drosophila* complex traits, including sleep (Huang *et al.* 2012; Swarup *et al.* 2012); 2272 known *D. melanogaster* genes are grouped with the gene ontology term “reproduction,” which includes, but is not limited to, genes involved in ovariole number development (Attrill *et al.* 2016). There are 17,727 genes total, so an unbiased survey of the entire genome would have a 12.8% chance of finding a gene involved in reproduction. We tested 46 mutations in 43 candidate genes and found 24 with significant effects on ovariole number (55.8%). The GWA therefore significantly enhanced our ability to discover candidate genes affecting female reproduction.

Most mutations and RNAi constructs did not show pleiotropic effects on other ovariole traits. Eight genes impacted ovariole asymmetry in addition to ovariole number, and none impacted ovariole number *CV_E_*. This finding is consistent with the low genetic correlation between ovariole number and ovariole number *CV_E_*, and suggests that different genes control ovariole number and its sensitivity to the environment. This differs from sleep traits for which the genetic architectures of trait means and their environmental sensitivities were largely shared (Harbison *et al.* 2013). Accordingly, some studies have observed little correlation between ovariole number and egg production (Robertson 1957; R′kha *et al.* 1997) or multigenerational fitness (Wayne *et al.* 1997), which may reflect the sensitivity of ovariole number to environmental factors such as temperature (Cohet and David 1978; Hodin and Riddiford 2000; Sarikaya *et al.* 2012; Klepsatel *et al.* 2013a,b), or food availability and nutrition (Hodin and Riddiford 2000; Tu and Tatar 2003; Wayne *et al.* 2006; Bergland *et al.* 2008). Additional work is needed to determine what factors influence these relationships.

Fewer variants were associated with ovariole number than with asymmetry and ovariole number *CV_E_*. This was not anticipated as ovariole number had greater broad-sense heritability than ovariole asymmetry and ovariole number *CV*_E_. However, broad-sense heritability, as we have calculated it here, encompasses all sources of genetic variation, including both dominance and epistatic effects (Falconer and Mackay 1996). Our result suggests that the contribution of additive genetic variance to ovariole number may be low. Furthermore, small, random fluctuations in bilateral characters ordinarily are due to sensitivity of genotypes to random environmental perturbations (Van Valen 1962). Thus, variation in ovariole number asymmetry likely indicates differences in canalization among DGRP genotypes. In this way, ovariole asymmetry and ovariole number *CV_E_* are similar traits: both measure variation in ovariole number among genetically identical flies. The genes implicated in variation in these traits comprise the genetic component of environmental variability (Harbison *et al.* 2013).

Even so, six genes are predicted by GWAS to impact both traits. These include *crossveinless c (cv-c)*—a component of the EGFR pathway implicated in tissue morphogenesis (Brodu and Casanova 2006)—and the ecdysone receptor (*EcR*). *EcR* plays an important role in ovary development (Hackney *et al.* 2007; Luo *et al.* 2015). It also has been shown by another study (Mendes and Mirth 2016) to affect plasticity in *Drosophila* ovariole number. Hormone pathways serve as proximate mechanisms underlying phenotypic plasticity across species (Zhou *et al.* 2007; Sommer and Ogawa 2011) (reviewed in Lema and Kitano (2013)). This is achieved through modulating the expression of downstream genes that affect tissue development (Markey *et al.* 2003). Interestingly, *EcR* also has been linked to phenotypic plasticity in butterfly eyespots (Monteiro *et al.* 2015). Thus, it may underlie plasticity more broadly.

In addition, we examined the GWA results for evidence of pleiotropic genes affecting both sleep and ovariole number traits. While no genetic correlation between ovariole number and sleep existed, and the overlap between polymorphisms common to each was low, there were 344 genes common to both studies. Recent work has argued that the partitioning of genetic variance into components at the phenotype level does not necessarily reflect underlying gene action, which may explain why we were able to find overlapping genes (Huang and Mackay 2016). The GWAS for ovariole number traits identified 943 unique candidate genes, 5.3% of the number of genes in the fly genome (17,727). We would expect 5.3% of the 3628 genes found in the sleep GWA to overlap by random chance, or 192 genes. Thus, the 344-gene overlap we observed between sleep and ovariole number is higher than would be expected by random chance. We confirmed the effects on both sleep and ovariole number for seven genes. Little LD existed among polymorphisms in these seven genes, implying differences in the forces maintaining them in nature. Our results are analogous to a previous study of *Catecholamines up* (*Catsup*), in which different polymorphic variants were significantly associated with lifespan, bristle number, startle response, and starvation resistance—though the genetic correlations among these traits were low (Carbone *et al.* 2006). Population genetics tests suggested that the polymorphisms in *Catsup* associated with different traits evolved independently of one another (Carbone *et al.* 2006). The same scenario may also be true for these seven genes, suggesting that independent evolution of polymorphisms for complex traits may be more widespread. Variation among complex traits would then be maintained by networks of polymorphisms for each trait responding to environmental conditions. Alternatively, if genes common to both traits interact with the environment, higher genetic correlations may exist under different environmental conditions.

## Supplementary Material

Supplemental material is available online at www.g3journal.org/lookup/suppl/doi:10.1534/g3.117.042390/-/DC1.
