# Supplementary material for: The Genetic Architecture of Ovariole Number in Drosophila melanogaster: Genes with Major, Quantitative, and Pleiotropic Effects
Source: G3 (Bethesda). 2017 May 26;7(7):2391–403. doi: 10.1534/g3.117.042390 (PMC5499145; doi:10.1534/g3.117.042390)
Supplement: Supplementary file 1 [file 2391FileS1.ppt]

## Slide 1
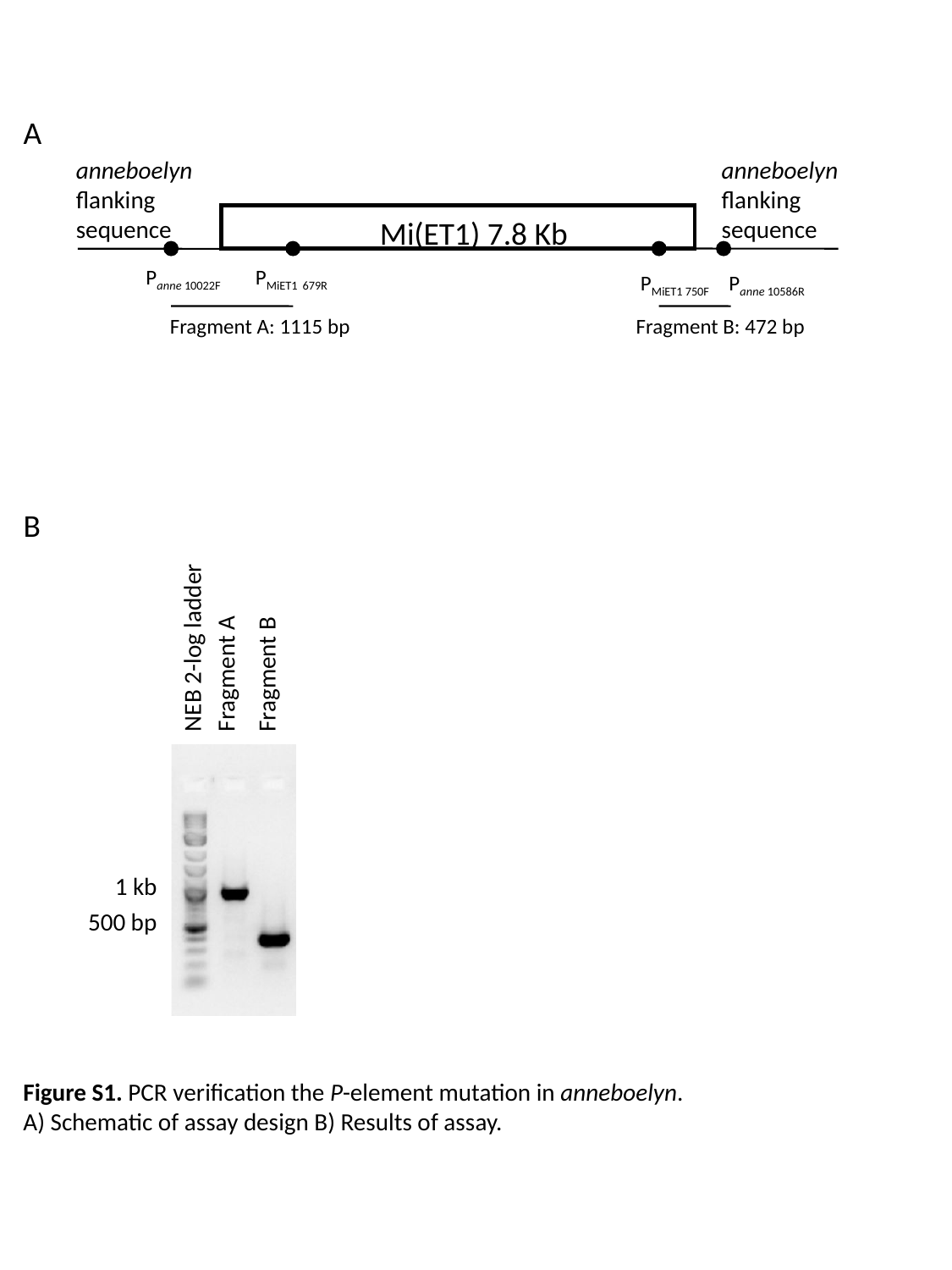

A
anneboelyn
flanking sequence
anneboelyn
flanking sequence
Mi(ET1) 7.8 Kb
Panne 10022F
PMiET1 679R
PMiET1 750F
Panne 10586R
Fragment A: 1115 bp
Fragment B: 472 bp
B
NEB 2-log ladder
Fragment A
Fragment B
1 kb
500 bp
Figure S1. PCR verification the P-element mutation in anneboelyn.
A) Schematic of assay design B) Results of assay.

## Slide 2
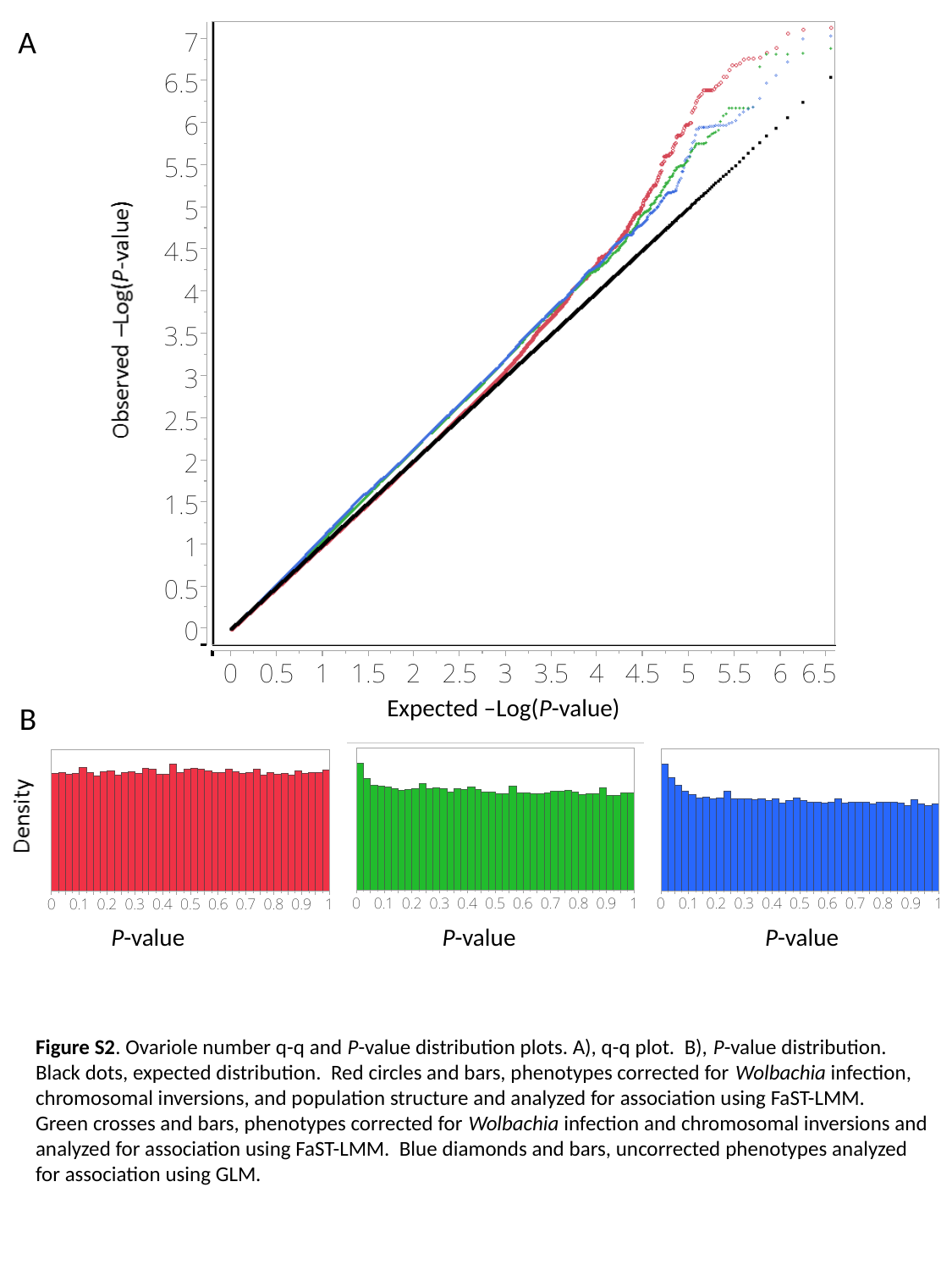

A
Expected –Log(P-value)
B
P-value
P-value
P-value
Figure S2. Ovariole number q-q and P-value distribution plots. A), q-q plot. B), P-value distribution. Black dots, expected distribution. Red circles and bars, phenotypes corrected for Wolbachia infection, chromosomal inversions, and population structure and analyzed for association using FaST-LMM. Green crosses and bars, phenotypes corrected for Wolbachia infection and chromosomal inversions and analyzed for association using FaST-LMM. Blue diamonds and bars, uncorrected phenotypes analyzed for association using GLM.

## Slide 3
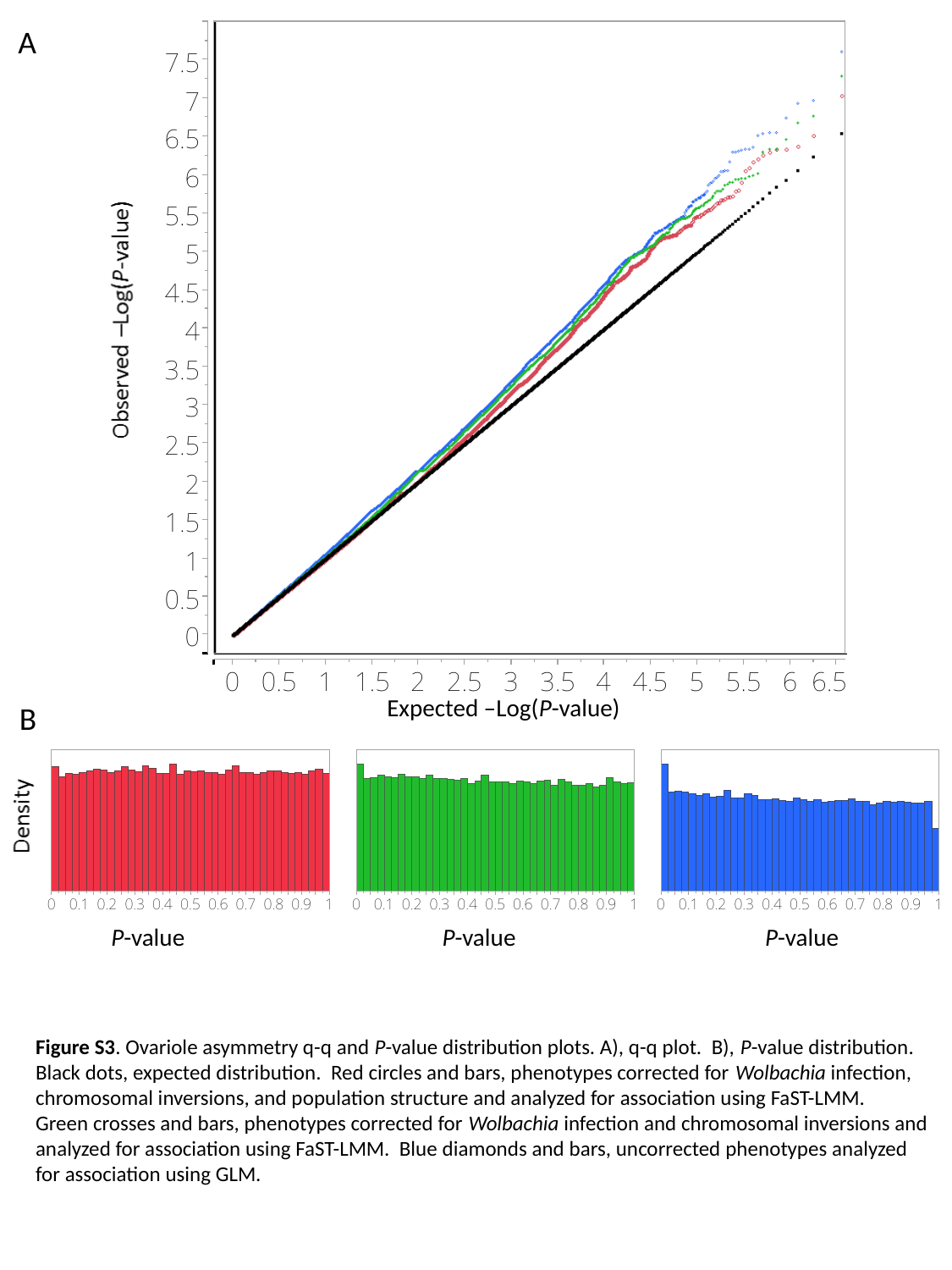

A
Expected –Log(P-value)
B
P-value
P-value
P-value
Figure S3. Ovariole asymmetry q-q and P-value distribution plots. A), q-q plot. B), P-value distribution. Black dots, expected distribution. Red circles and bars, phenotypes corrected for Wolbachia infection, chromosomal inversions, and population structure and analyzed for association using FaST-LMM. Green crosses and bars, phenotypes corrected for Wolbachia infection and chromosomal inversions and analyzed for association using FaST-LMM. Blue diamonds and bars, uncorrected phenotypes analyzed for association using GLM.

## Slide 4
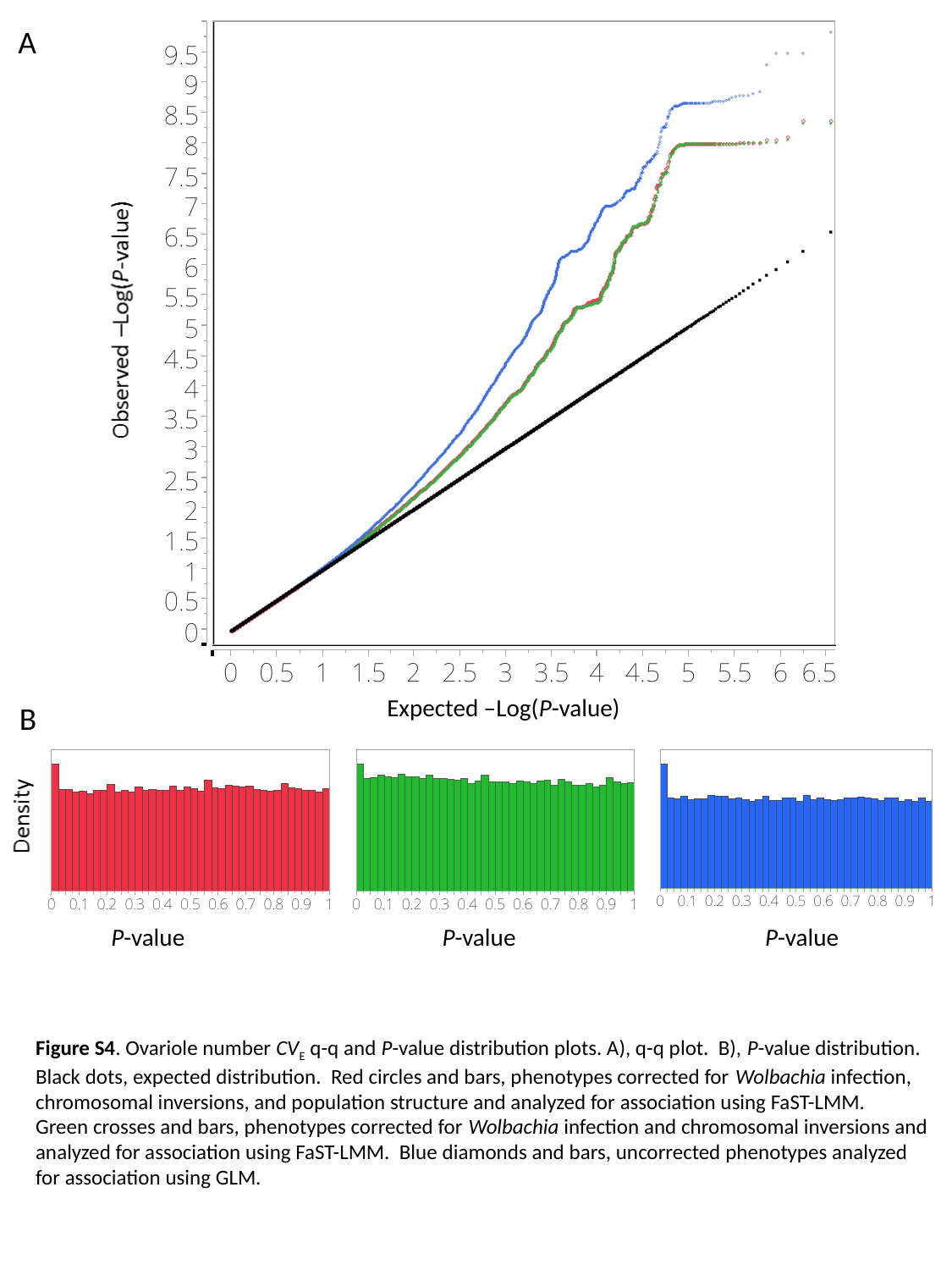

A
Expected –Log(P-value)
B
P-value
P-value
P-value
Figure S4. Ovariole number CVE q-q and P-value distribution plots. A), q-q plot. B), P-value distribution. Black dots, expected distribution. Red circles and bars, phenotypes corrected for Wolbachia infection, chromosomal inversions, and population structure and analyzed for association using FaST-LMM. Green crosses and bars, phenotypes corrected for Wolbachia infection and chromosomal inversions and analyzed for association using FaST-LMM. Blue diamonds and bars, uncorrected phenotypes analyzed for association using GLM.

## Slide 5
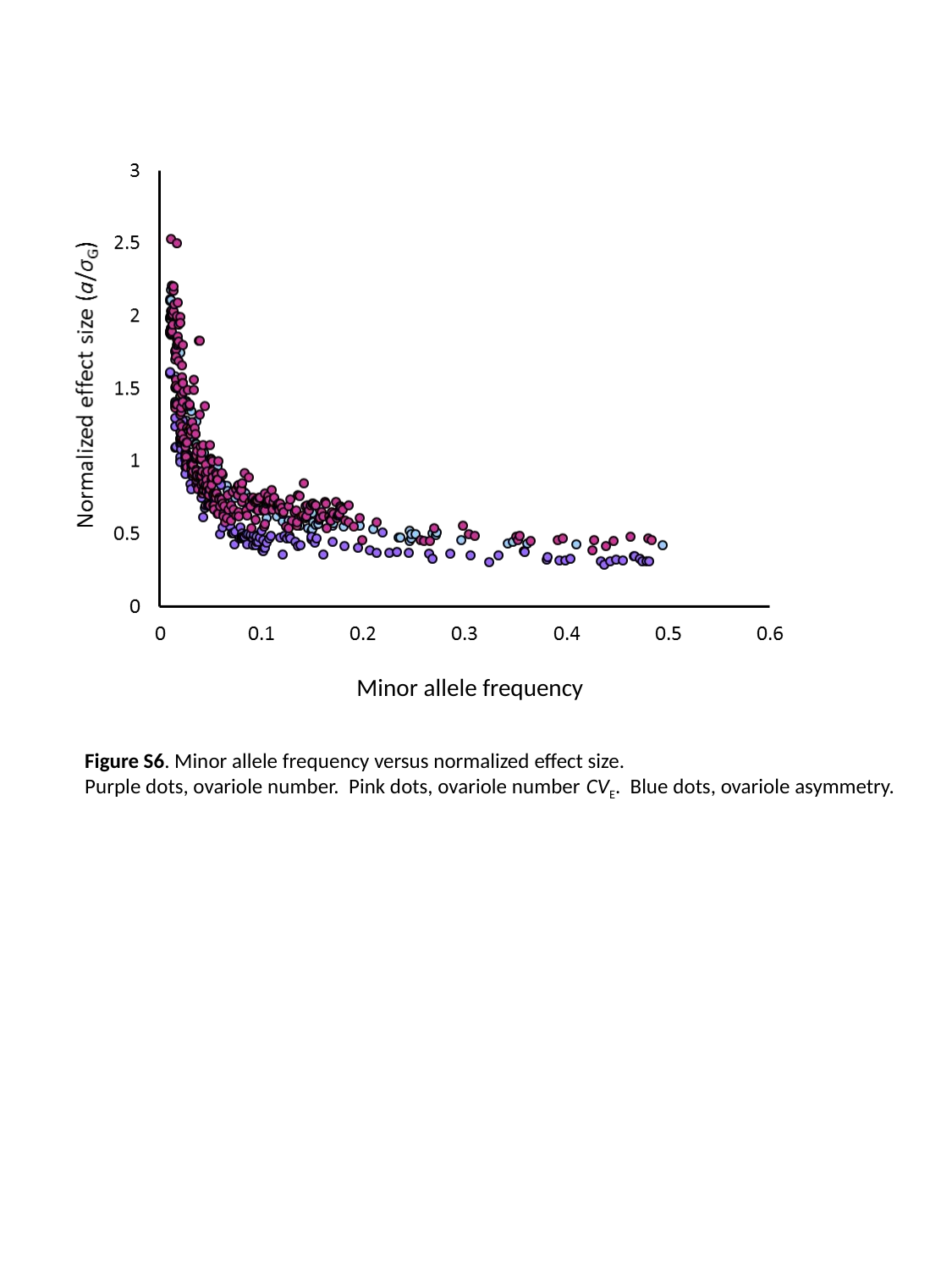

Minor allele frequency
Figure S6. Minor allele frequency versus normalized effect size.
Purple dots, ovariole number. Pink dots, ovariole number CVE. Blue dots, ovariole asymmetry.

## Slide 6
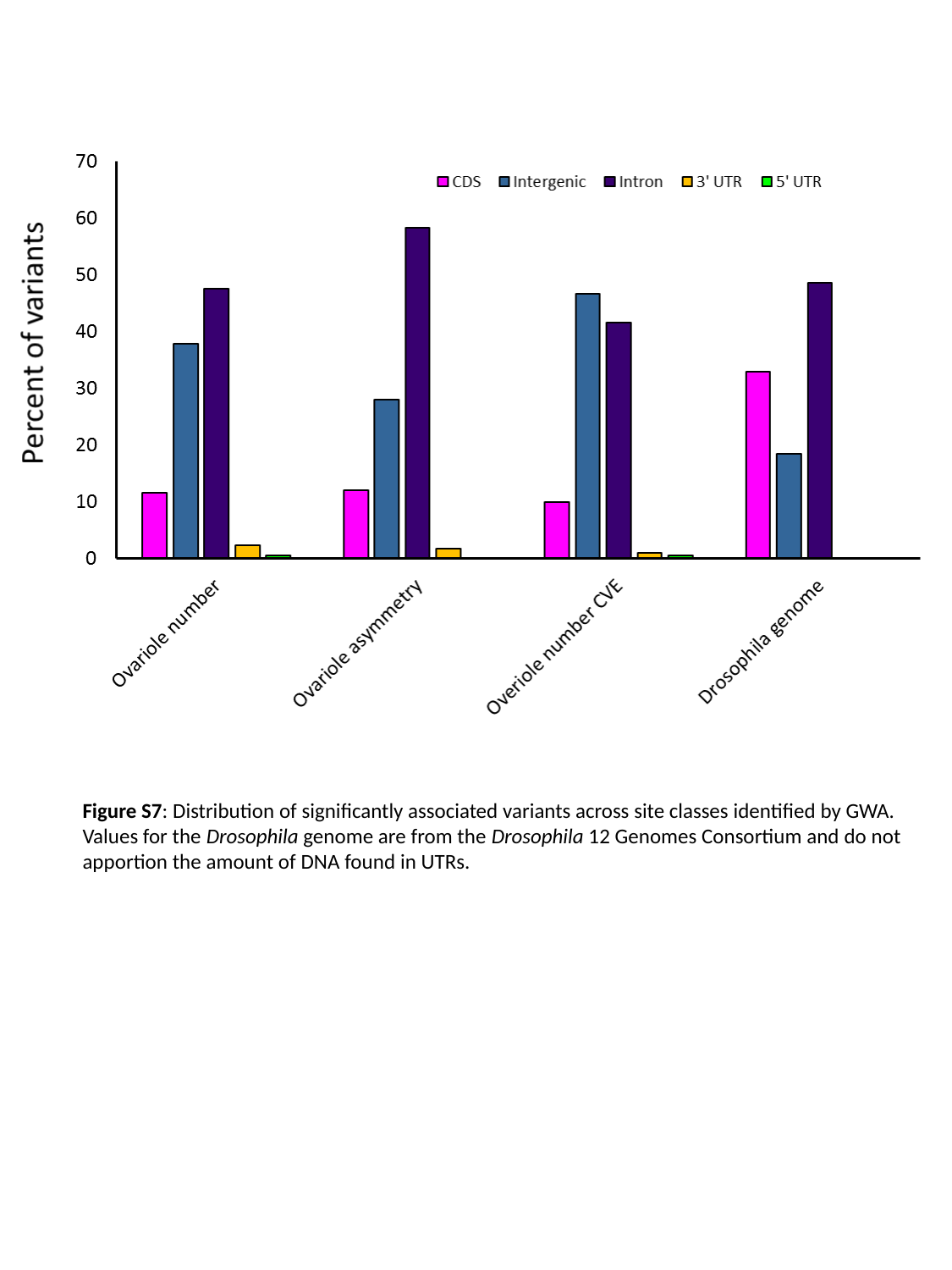

Figure S7: Distribution of significantly associated variants across site classes identified by GWA. Values for the Drosophila genome are from the Drosophila 12 Genomes Consortium and do not apportion the amount of DNA found in UTRs.
