# Supplementary figures and images for: The Genetic Architecture of Ovariole Number in Drosophila melanogaster: Genes with Major, Quantitative, and Pleiotropic Effects
Source: G3 (Bethesda). 2017 May 26;7(7):2391–403. doi: 10.1534/g3.117.042390 (PMC5499145; doi:10.1534/g3.117.042390)

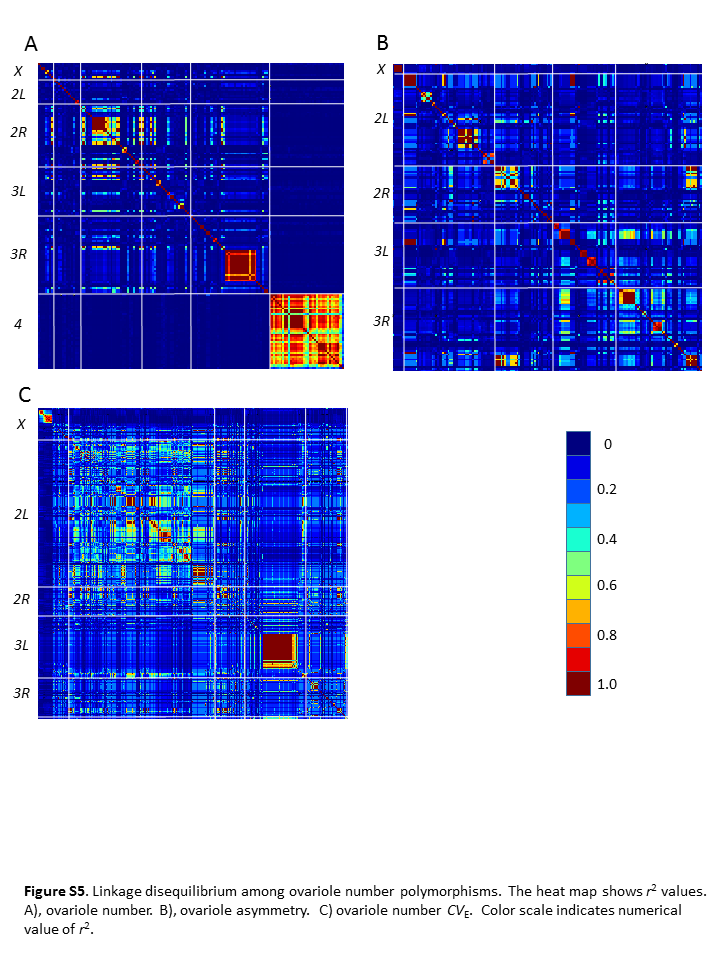

Supplement: Supplementary file 3 [file 2391FigureS5.tif]
